# Identification of RUNX1 and IFNGR2 as prognostic-related biomarkers correlated with immune infiltration and subtype differentiation of low-grade glioma

**DOI:** 10.17305/bjbms.2022.8086

**Published:** 2023-05-01

**Authors:** Xia Zhang, Hongyu Chu, Yuan Cheng, Jie Ren, Wei Wang, Xicheng Liu, Xiaodong Yan

**Affiliations:** 1 Department of Physiology and Pathophysiology, School of Basic Medical Sciences, Capital Medical University, Beijing, China; 2 Beijing Bo’ai Hospital, China Rehabilitation Research Center, Beijing, China; 3 School of Rehabilitation, Capital Medical University, Beijing, China; 4 Department of Immunology, School of Basic Medical Sciences, Capital Medical University, Beijing, China; 5 School of Clinical Medicine, Jining Medical University, Jining, China

**Keywords:** Runt-related transcription factors (RUNXs), low-grade glioma (LGG), interferon-gamma receptor 2 (IFNGR2), tumor microenvironment (TME), immune infiltration, immune checkpoints

## Abstract

Immune cell infiltration occurs in the tumor microenvironment (TME) and influences cancer progression through interaction with tumor cells. Runt-related transcription factors (RUNXs), including RUNX1–3, are the master regulators of development and differentiation and are all important to the development of immune cells. However, the role of RUNXs in the immune cells of TME remains unclear. In this study, we first used online related databases and related low-grade glioma (LGG) data from TCGA and CGGA to conduct bioinformatics analysis. The analysis confirmed that RUNXs were significantly and positively correlated with immune infiltration in multiple tumors, especially in LGG and there was the highest correlation between RUNXs and the progress and prognosis of LGG. Furthermore, the functional enrichment analysis revealed that RUNXs might be involved in the inflammatory and immune responses of the biological processes, and RUNXs were tightly associated with the multiple immune checkpoint molecules. Subsequent results confirmed that RUNX1, as an independent prognostic factor for LGG, may target interferon-gamma receptor 2 (IFNGR2) to regulate glioma cell proliferation, invasion, and migration. Besides, we also found that the expression levels of RUNX1 and IFNGR2 were significantly reduced, and their correlation was enhanced in the IDH-mutant subtype. Patients with a high expression of RUNX1 and/or IFNGR2 (HH/H) in the IDH-mutant subtype showed poorer prognosis and significantly increased infiltration of M2 macrophages. This finding implied the possible key role of RUNX1 in the differentiation of IDH mutant subtypes as well as in the formation of TME infiltration signatures by monitoring IFNGR2.

## Introduction

Glioma is the most common primary tumor in the central nervous system. According to the 2021 new classification specified by the World Health Organization (WHO), adult-type diffuse gliomas can mainly be categorized into three histological subtypes: (1) Astrocytoma, (2) Oligodendroglioma, and (3) Glioblastoma [1]. Low-grade glioma (LGG) belongs to a large class of gliomas with great intrinsic heterogeneity in terms of the biological behavior of a tumor [2], which includes diffuse low-grade and intermediate-grade gliomas (WHO grades 2 and 3) [3, 4]. LGG can be classified into several types, such as IDH wild-type (WT), IDH mutation alone (IDH-noncodel), and IDH mutation with 1p/19q codeletion (IDH-codel). However, the effect of treatment significantly varies with the different subtypes [1, 5]. In recent years, in-depth studies of the tumor microenvironment (TME) have made immunotherapy an important approach in treating diverse tumor types, including gliomas [6]. Moreover, some studies have confirmed a close association between different immune cell infiltrations and the cumulative survival rate of LGG [7]. Because the current research on LGG has not been researched in depth, the treatment choice is affected by several factors, and the links between clinical diagnosis and treatment remain controversial. Therefore, there is an urgent need for a new high-accuracy immunological biomarker to predict the prognosis of different subtypes of LGG so as to serve as a standard reference for immunotherapy.

Dysregulation of transcription factors induced by gene mutations is the key factor contributing to the occurrence of cancer [8]. Runt-related transcription factors (RUNXs), such as RUNX1, RUNX2, and RUNX3, have been evolutionarily conserved across simple to complex organisms and are the key to the process of development and biology in mammals [9]. RUNX encodes the alpha subunit of polyomavirus enhancer binding protein 2/core binding factor (PEBP2/CBF) transcription factor [10], which is composed of DNA binding and non-DNA binding subunits. All RUNXs contain transcriptional activation and inhibition domains (ID), which are located at the C-terminus [11]. They also contain scattered RUNX binding sites that may be responsible for feedback regulation among RUNXs [12]. Some past studies have demonstrated that RUNXs are the master regulators of development and differentiation and frequently dysregulated in cancer [13–16]. RUNX1, RUNX2, and RUNX3 have their unique functions that contribute to the regulation of development, and they are all important to the development of immune cells [17, 18]. It is already well established that the immune microenvironment influences tumor progression through the interactions with tumor cells [19], but the role of RUNXs in the immune infiltration of TME remains unclear.

In our past studies, we first conducted a comprehensive assessment of the differential expression of RUNXs in various types of human cancers, analyzed the correlation between RUNXs expression and immune infiltration in the TME of different solid tumors, assessed the prognostic significance of their expression and the closer co-relationship between RUNXs and immune infiltration, and verified the prognosis of LGG. We further investigated the relationship between RUNXs and common immune checkpoints in LGG. The independent prognostic value of RUNX1, RUNX2, and RUNX3 in LGG was analyzed by Multivariate Cox regression analysis and then further verified with reference to the specific data of The Cancer Genome Atlas (TCGA) and Chinese Glioma Genome Atlas (CGGA). Finally, RUNX1 and its target gene interferon-gamma receptor 2 (IFNGR2) were associated with growth, apoptosis, invasion, and migration of LGG cells, as well as immune infiltration and poor prognosis of LGG. Further analysis clarified that RUNX1 may be involved in the refinement of the LGG IDH mutation subtypes and immune infiltration by regulating IFNGR2.

The present results provide novel insights into the functional role of RUNXs in LGG. Accordingly, we conclude that RUNX1 represents the family of RUNXs as an independent risk factor for LGG and may serve as a molecular target for IDH mutant subtype refinement and immunotherapy. Thus, there is a need to highlight the potential mechanistic basis for RUNXs to influence immune cell–tumor interactions.

## Materials and methods

### Oncomine database analysis

As a comprehensive database and an integrated data mining platform, the Oncomine database (www.oncomine.org) contains 715 gene expression datasets from 86,733 samples [20]. Therefore, we employed this database to assess the expression of RUNXs in different cancer types.

### TIMER database analysis

TIMER is a comprehensive website (https://cistrome.shinyapps.io/timer/) [21] designed for analyzing and visualizing the association between numerous variables and immune infiltrates. These immune infiltrates include B cells, CD4^+^/CD8^+^ T cells, macrophages, neutrophils, and dendritic cells via gene modules. This database contains 10,897 samples covering 32 cancer types from TCGA. A series of analyses on the expression of RUNXs in different tumors and their relationship with the abundance of immune infiltrates and patient prognosis were conducted in this study.

### GEPIA database analysis

GEPIA (http://gepia.cancer-pku.cn/) [22], a new online database designed to perform a variety of standard analyses of gene expression, is associated with cancer and contains sequencing expression data from 9736 tumor samples of 33 cancer types and 8587 normal samples in the TCGA and GTEx datasets. Therefore, we employed this database to assess the expression of RUNXs in different tumors and their correlation with patient prognosis.

### UALCAN analysis

UALCAN (http://ualcan.path.uab.edu) is a comprehensive, user-friendly, and interactive Web resource used for analyzing cancer transcriptome, proteomics, and patient survival data using TCGA gene expression data and clinical data from 33 cancer types [23]. These included the analysis of gene expression data in different tumor sub-groups based on individual cancer stages, tumor grade, or other clinicopathological features.

### TISIDB analysis

TISIDB is a web portal for tumor and immune system interaction, which integrates multiple types of data resources in oncoimmunology [24]. The associations between RUNXs genes and immune features, such as lymphocytes, MHC molecules, immune inhibitors, immunostimulators, and immune subtype, were pre-calculated for 30 TCGA cancer types. The *P*-value and Spearman’s correlation coefficients (rho) were calculated automatically. Availability and implementation: http://cis.hku.hk/TISIDB.

### LGG patient datasets

We downloaded the LGG patient datasets, with gene expression profiles and clinical information from the publicly available TCGA database via the Cbioportal website (https://www.cbioportal.org/) [25] and CGGA [26] (http://www.cgga.org.cn/), respectively. In addition to the online public database, a total of 696 patients with confirmed LGG (TCGA: 514 patients; CGGA: 182 patients) were included in this analysis (Tables S1 and S2). In order to meet the evaluation needs of different grades of patients’ RUNXs expression differences, the inclusion criteria we analyzed in the two databases include 1) not only grade 2 but also grade 3 patients defined by the 2021 WHO Classification and 2) patients with complete clinical and transcriptional data.

### PrognoScan database analysis

The PrognoScan database (http://dna00.bio.kyutech.ac.jp/PrognoScan/) is a new database for meta-analysis of the prognostic value of genes, which is designed to search the relation between gene expression and patient prognosis, such as overall survival (OS) and disease free survival (DFS), across a large collection of publicly available cancer microarray datasets [27]. Therefore, we used this database to analyze the relationship between RUNXs expression and patient prognosis.

### LinkedOmics analysis

The LinkedOmics database (http://www.linkedomics.org/login.php) is publicly available portal that includes multi-omics data from all 32 TCGA cancer types and 10 Clinical Proteomics Tumor Analysis Consortium (CPTAC) cancer cohorts [28]. The Web application has three analytical modules: LinkFinder, LinkInterpreter, and LinkCompare. The LinkFinder module was used to study genes differentially expressed in correlation with RUNXs in the TCGA LGG cohort (*n* ═ 516). Results were analyzed statistically using Spearman’s correlation coefficient. Data from the LinkFinder results were signed and ranked, and were used to perform analyses of Gene Ontology (GO) (Biological Process, BP; Cellular Component, CC; and Molecular Function, MF), Kyoto Encyclopedia of Genes and Genomes (KEGG) pathways enrichment. The rank criterion was *P* < 0.01, FDR < 0.02.

### GeneMANIA analysis

GeneMANIA (http://www.genemania.org) is a comprehensive and public web interface using a very large set of functional association data, including protein and genetic interactions, pathways, co-expression, co-localization, and protein domain similarity [29]. The website can construct protein–protein interaction (PPI) network and find new members of a pathway or complex, find additional genes you may have missed in your screen or find new genes with a specific function. We used GeneMANIA to visualize the genes networks and predict function of the top 20 genes positively correlated with RUNX1, RUNX2, and RUNX3 obtained from the LinkedOmics analysis.

### Cell culture and transfection

Two human glioma (SW1088 and HS683) cell lines were derived from ATCC (Manassas, VA, USA). Leibovitz’s L-15 medium (Invitrogen, 11415064) with penicillin–streptomycin solution (NCM Biotech, C100C5), and 10% FBS (Invitrogen, 10099141C) was used as the culture medium of SW1088 cells. Dulbecco’s modified Eagle’s medium (DMEM, Gibco, 10569010) with penicillin–streptomycin solution, and 10% FBS was used as the culture medium of HS683 cells. The cells were cultured in an incubator at 37 ^∘^C and 5% CO_2_. When the cell fusion rate reaches more than 80%, the cells are used for experimental intervention. The RUNX1 Human shRNA Plasmid Kit (Locus ID 861) was purchased from OriGene Technologies (Wuxi, China). RUNX1-Human, 2 unique 29mer shRNA (Cat No: TL309685) targets are shown as follows: TL309685C# CAAGTCGCCACCTACCACAGAGCCATCAA; TL309685D# TGCCTACGCACTGGCGCTGCAACAAGACC. The RUNX1 over-expression plasmid was constructed by OBiO Technology Co., Ltd (Shanghai, China). According to the instructions, plasmid above was transfected into human glioma cell lines through Lipofectamine 3000 (Invitrogen, L3000008).

### CCK-8 detection

Two human glioma (SW1088 and HS683) cell lines were inoculated into 96-well plates at a density of 1 ×10^3^ cells/well, and then the cells were cultured in a CO_2_ incubator at 37 ^∘^C. According to the instructions of the CCK-8 cell proliferation and cytotoxicity detection kit (Solarbio, CA1210) and different experimental groups, after the cells were subjected to knockdown and overexpression treatment, the cells were placed in the CO_2_ incubator for the specified time (0, 24, 48, 72 h). Add 10 µL CCK-8 solution to each well of the 96 well plate. The culture plates were incubated in the incubator for 30 minutes. Mix gently before reading 96 well plate. Absorbance at 450 minutes was measured using a microplate reader.

### Cell invasion assay

Two human glioma (SW1088 and HS683) cell lines were inoculated into 24-well plates (1 × 10^4^ cells/well). Different groups of cells were pretreated according to the experimental conditions. Put Transwell chamber into 24-well plate, add 60 µL diluted matrigal glue (dilution ratio: 1:8), and incubate it in incubator for 4–5 hours. After the upper chamber gel solidifies, absorb the residual liquid, then add 100 µL serum-free medium, and then put it into the incubator again for incubation for 20 minutes. After cells were digested, cell suspension was prepared with serum free medium and counted with blood cell counting plate. 1 × 10^4^ cells were inoculated into the upper chamber, medium containing 10% FBS was inoculated into the lower chamber, and incubated at 37 ^∘^C and 5% CO_2_ for 24 hours. Remove the culture medium and fix the cells with 4% paraformaldehyde for 15 minutes. The cells were stained with crystal violet for 20 minutes, washed with phosphate buffer saline (PBS), and imaged with microscope.

### Wound healing assay

Two human glioma (SW1088 and HS683) cell lines were inoculated into 6-well plates (5 × 10^5^ cells/well). Different groups of cells were pretreated according to the experimental conditions. First, place the ruler on the bottom of the dish and mark a central vertical line and five horizontal lines with a black pen, and then use a sterile pipette tip to draw lines in the dish compared to the marked lines. Finally, cells were washed three times with PBS to remove streaked cells and replaced with fresh medium. Cells were incubated in a 37 ^∘^C, 5% CO_2_ incubator, and imaged with microscope (Nikon ECLIPSE 80i, Japan) for the indicated times (0, 24, 48, 72 hours).

### Apoptosis detection

Two human glioma (SW1088 and HS683) cell lines were inoculated into 6-well plates, and then the cells were cultured in a CO_2_ incubator at 37 ^∘^C. According to different experimental groups, cells were subjected to knockdown and overexpression treatment. Then, the subsequent experiments were carried out according to the instructions of annexin V-FITC/PI apoptosis detection kit (Solarbio, CA1020). First, collect the cell culture solution into a new tube, wash the cells with PBS, and then add EDTA-free trypsin to digest the cells. Subsequently, the collected cell culture medium was added to terminate the digestion, and the cells were collected into the centrifuge tube. After centrifugation at 1000 *g* for 5 minutes, the supernatant was discarded and the cells were collected. The cells were resuspended with PBS and counted. Resuspended cells (5× 10^4^) was centrifuged again at 1000 *g* for 5 minutes, the supernatant was discarded, and 195 µL annexin V-FITC binding solution was used to resuspend the cells. Then add 5 µL of annexin V-FITC and mixed well. Finally, add 10 µL of propidium iodide (PI) staining solution and mixed well. After incubation at room temperature for 15 minutes in the dark, apoptosis was detected by flow cytometry.

### Cell cycle assay

Two human glioma (SW1088 and HS683) cell lines were inoculated into 6-well plates (10 × 10^5^ cells/well). Different groups of cells were pretreated according to the experimental conditions. After discarding the medium, trypsinize the cells with EDTA-free trypsin, add cell culture medium to terminate the digestion, and collect the digested cells into a centrifuge tube. The cells were centrifuged at 1500 *g* for 5 minutes, and the supernatant was discarded. Add 5 mL of precooled 70% ethanol, mix well, and fix overnight at 4 ^∘^C. The cells were washed with PBS, centrifuged at 1500 *g* for 5 minutes and collected, and the operation was repeated twice. Add 500 𝜇L solution (100 g/mL RNase A, 0.2% Triton X-100) to the cell precipitation, resuspend the cells, and incubate at 37 ^∘^C for 30 minutes. The cells were washed twice with 5 mL PBS and stained with 400 𝜇L PI solution for 10 minutes in dark. The cell suspension was filtered with a 300 mesh cell sieve. The cells were centrifuged at 1500 *g* for 5 minutes, and the supernatant was discarded. Add 5 mL PBS to wash cells to remove excess PI, and use 100 𝜇L PBS resuspends cells. Finally, flow cytometry was used for detection.

### Western blot assay

Two human glioma (SW1088 and HS683) cell lines were inoculated into 6-well plates (5× 10^5^ cells/well). Different groups of cells were pretreated according to the experimental conditions. Remove the cell culture medium and wash the cells with pre-cooled PBS for three times. Cell samples were lysed with RIPA lysis buffer (Applygen Technology, C1053) supplemented with PMSF (Beyotime Biotechnology, ST506) and protease inhibitor (Roche, 04693132001) at 37 ^∘^C for 1 hour. The lysed cell samples were centrifuged at 12000 *g* at 4 ^∘^C for 20 minutes. After collecting the supernatant of cell samples, the protein concentration was determined by BCA protein determination kit (Thermo Fisher Scientific, 23225). The supernatant was boiled at 99 ^∘^C for 10 minutes in protein loading buffer (ROBY, RBU114-2). The protein samples were loaded into 12% SDS-PAGE, and the target bands were separated by running buffer (Applygen Technology, B1005). Then, the target protein was transferred from SDS-PAGE to PVDF membrane (Millipore, IPVH00010) through transfer buffer (Solarbio, D1060). The membranes were incubated in a blocking solution containing 5% nonfat powdered milk (Solarbio, D8340) at RT for 1 hour. After washing with tris-buffered saline tween-20 (TBST) for three times, the samples were detected with the indicated primary antibody, and incubated overnight at 4 ^∘^C. The membrane was washed with TBST for three times and incubated with HRP linked secondary antibody at RT for 1 hour. After washing with TBST for three times, the samples were detected by chemiluminescence (NCM Biotech, P10300) and visualized by Amersham imager 600 (General Electric Company, USA). β-actin is used as the loading control. The antibody information used in the experiment is as follows: Anti-RUNX1 (Abcam, ab23980, dilution ratio: 1:750); Anti-IFNGR2 (Proteintech, 10266-1-AP, dilution ratio: 1:750); Anti-β-actin (Proteintech, 60008-1-Ig, dilution ratio: 1:5000); and Anti-rabbit/mouse IgG, HRP-linked antibody (Cell Signaling Technology, 7074s/7076s, dilution ratio: 1:5000).

### Real-time quantitative PCR

Two human glioma (SW1088 and HS683) cell lines were transfected in different group, then the total RNA was extracted with the TRIzol reagent (Invitrogen Life Technologies, Chicago, IL, USA). The specific primers (Qiagen, Hilden, Germany) and the SYBR-Green PCR Master Mix Kit (Takara, Japan) were used to RT-qPCR on the ABI 7500 System (Applied Biosystems, Foster City, CA, USA). β-actin was used for normalizing the gene expression. All primers used in this study were synthesized by Tsingke Biotechnology Co., Ltd (Xian, China). The primers used were listed in Table S3.

### Statistical analysis

Data obtained from TCGA and CGGA merged and are processed by R3.5.3. The correlations between the clinical information and RUNXs expression were analyzed using logistic regression. Moreover, multivariate Cox analysis was used to evaluate the influence of RUNXs expression and other clinicopathological factors (age, gender, and subtype or purity) on survival. *P*-value < 0.05 was set up as the cut-off criterion. Statistical analyses were performed by one-way ANOVA or *t*-tests using GraphPad Prism 6.01 software. The results are presented as the mean ± standard error of the mean (SEM).

## Results

### Assessment of RUNXs (RUNX1-3) expression in a series of tumor and normal tissues

We first employed the Oncomine database and TIMER database, respectively, to assess the expression of RUNXs in different tumors and normal tissues. The analysis using the Oncomine database revealed that the expression of RUNX1, RUNX2, and RUNX3 was elevated in multiple tumors relative to those of the normal tissues.

**Figure 1.**
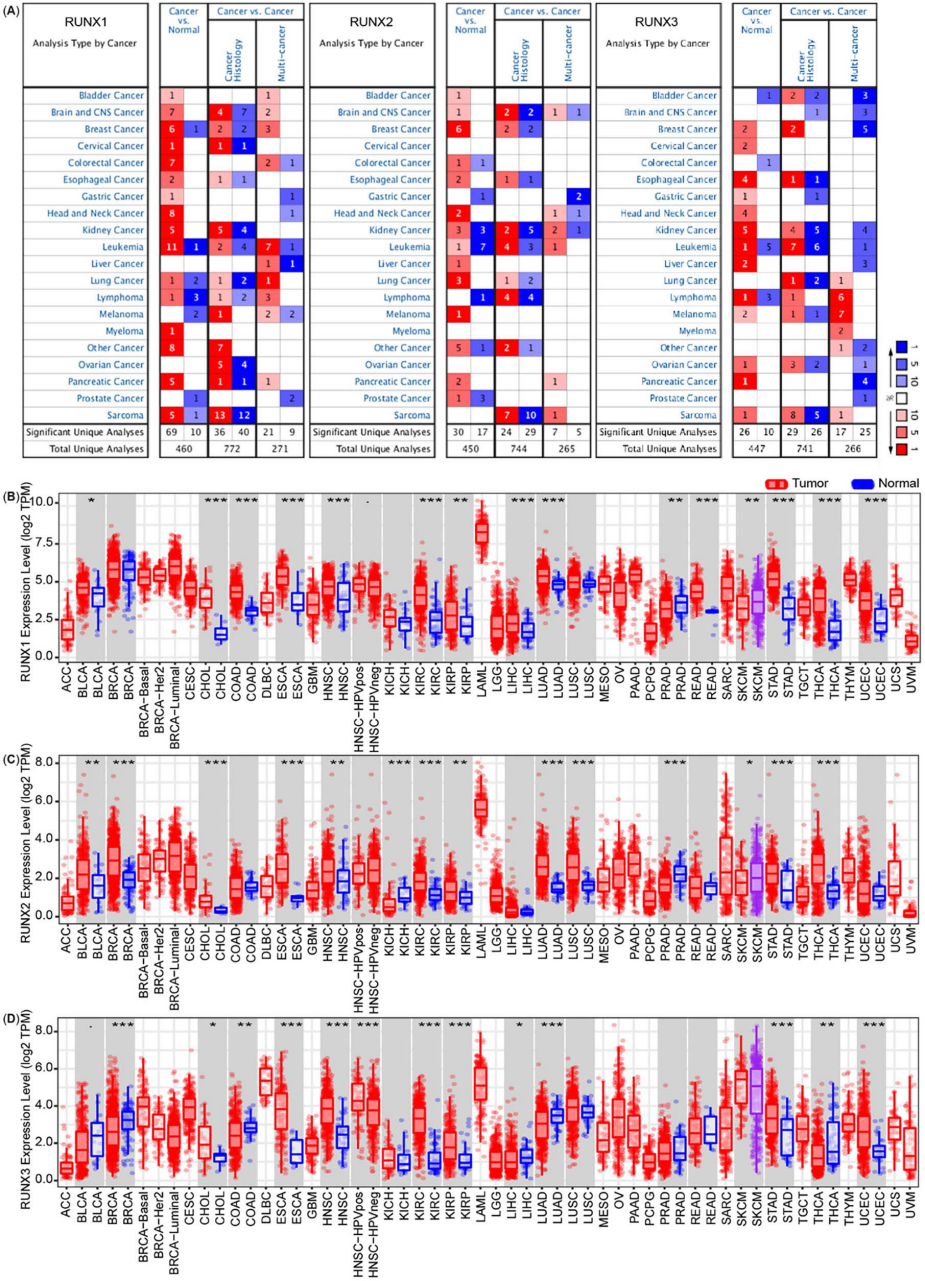
**Runt-related transcription factors (RUNX1, RUNX2, and RUNX3) expression levels in different types of human cancers.** (A) Increased or decreased RUNXs in datasets of different cancers compared with normal tissues in the Oncomine database. Red and blue refer to the number of datasets with statistically significant RUNXs mRNA overexpression or down-regulation, respectively. (*P*-value is 0.001, fold change is 2.0, and gene ranking of all.) (B)–(D) RUNXs expression levels in different tumor tissues and normal tissues in TIMER database (^*^P < 0.05, ^**^P < 0.01, ^***^P < 0.001). Red and blue refer to tumor tissue and normal tissue, respectively.

Compared with normal tissues, the RUNX1 expression increased in 15 cancers (bladder, brain, breast, cervical, colorectal, esophageal, gastric, head and neck, kidney, leukemia, lung, lymphoma, myeloma, pancreatic, and sarcoma cancers) and decreased in 7 cancers (breast, leukemia, lung, lymphoma, melanoma, prostate, and sarcoma cancers) (Figure 1A, left panel). Compared with normal tissues, the RUNX2 expression increased in 13 tumors (bladder, brain, breast, colorectal, esophageal, head and neck, kidney, leukemia, liver, lung, melanoma, pancreatic, and prostate cancers) and decreased in 6 tumors (colorectal, gastric, kidney, leukemia, lymphoma, and prostate cancers) (Figure 1A, middle panel). We also noted that the RUNX3 expression increased in 13 tumors (breast, cervical, esophageal, gastric, head and neck, kidney, leukemia, liver, lymphoma, melanoma, ovarian, pancreatic, and sarcoma cancers) and decreased in 4 tumors (bladder, colorectal, leukemia, and lymphoma cancers) relative to that in the normal tissues (Figure 1A, right panel).

The TIMER database was further used to estimate the expression of RUNX1, RUNX2, and RUNX3 in specific types of tumors. The analysis revealed that the expression of RUNXs changes in a variety of tumors, most of which showed an elevated expression trend. The expression of RUNX1 in 15 of 32 cancers was changed relative to that in the corresponding normal tissues, including increased expression in 13 cancers and decreased expression in 2 cancers (Figure 1B). The expression of RUNX2 in 14 of 32 cancers was also found to have modified relative to that in the corresponding normal tissues, which included increased expression in 11 cancers and decreased expression in 3 cancers (Figure 1C). The expression of RUNX3 in 12 of 32 cancers was found to be modified relative to that in the corresponding normal tissues, which included increased expression in 7 cancers and decreased expression in 5 cancers (Figure 1D). All of them were highly expressed in cholangiocarcinoma (CHOL), esophageal carcinoma (ESCA), head and neck squamous cell carcinoma (HNSC), kidney renal clear cell carcinoma (KIRC), kidney renal papillary cell carcinoma (KIRP), and stomach adenocarcinoma (STAD) than in the normal tissues.

We also used the GEPIA database to analyze the expression of RUNXs in different cancer types and normal tissues. The expression of RUNX1 was increased relative to that in the normal tissues in 17 of 33 cancers, showing a decrease only in 1 cancer (Figure S1A); the expression of RUNX2 was elevated relative to that in the normal tissues in 11 of 33 cancers and decreased in 2 cancers (Figure S1B), and the expression of RUNX3 was increased in 17 of 33 cancers and decreased in 2 cancers relative to that in the normal tissues (Figure S1C). All of them were highly expressed in ESCA, HNSC, KIRC, acute myeloid leukemia (LAML), pancreatic adenocarcinoma (PAAD), and STAD relative to that in the normal tissues.

Different members of RUNXs displayed different expression states in different tumors, with an overall upward trend in most tumors. This phenomenon suggests that the possible mechanism is also different, and it is worth further analysis.

### The expression of RUNXs (RUNX1-3) is correlated with immune infiltration in multiple tumors

Several studies have demonstrated that immune infiltration around solid tumors can act as an efficient independent survival predictor of different cancers [30–37]. The RUNXs has been best known for their critical roles in hematopoiesis, particularly during the development of T cells [17, 18]. However, there are a few reports about their role in the immune infiltration around the tumor.

Therefore, we next used the TIMER database to estimate whether RUNXs were associated with immune infiltration in 32 tumor types. As illustrated in the heat maps (Figure S2A–S2C), a negatively significant correlation was noted between RUNXs expression and the tumor purity in several tumor types; specifically in 19 tumor types for RUNX1, in 21 types for RUNX2, and in 23 types for RUNX3. A significant positive correlation was noted between RUNXs expression and B cell infiltration in several tumor types; specifically, in 17 types for RUNX1, in 18 types for RUNX2, and in 21 types for RUNX3. There were additional positive correlations between RUNXs and the levels of other immune cell infiltration in different tumor types. CD8^+^ T cell infiltration was recorded in 21 tumor types for RUNX1, in 18 types for RUNX2, and in 21 types for RUNX3. In addition, CD4^+^ T cell infiltration was noted in 20 types for RUNX1, in 19 types for RUNX2, and in 23 types for RUNX3. Macrophage infiltration was recorded in 22 tumor types for RUNX1, in 25 types for RUNX2, and in 18 types for RUNX3. Moreover, neutrophil infiltration was noted in 26 types for RUNX1, in 23 types for RUNX2, and in 25 types for RUNX3. Finally, dendritic cell infiltration was detected in 24 tumor types for RUNX1, in 24 types for RUNX2, and in 26 types for RUNX3.

**Figure 2.**
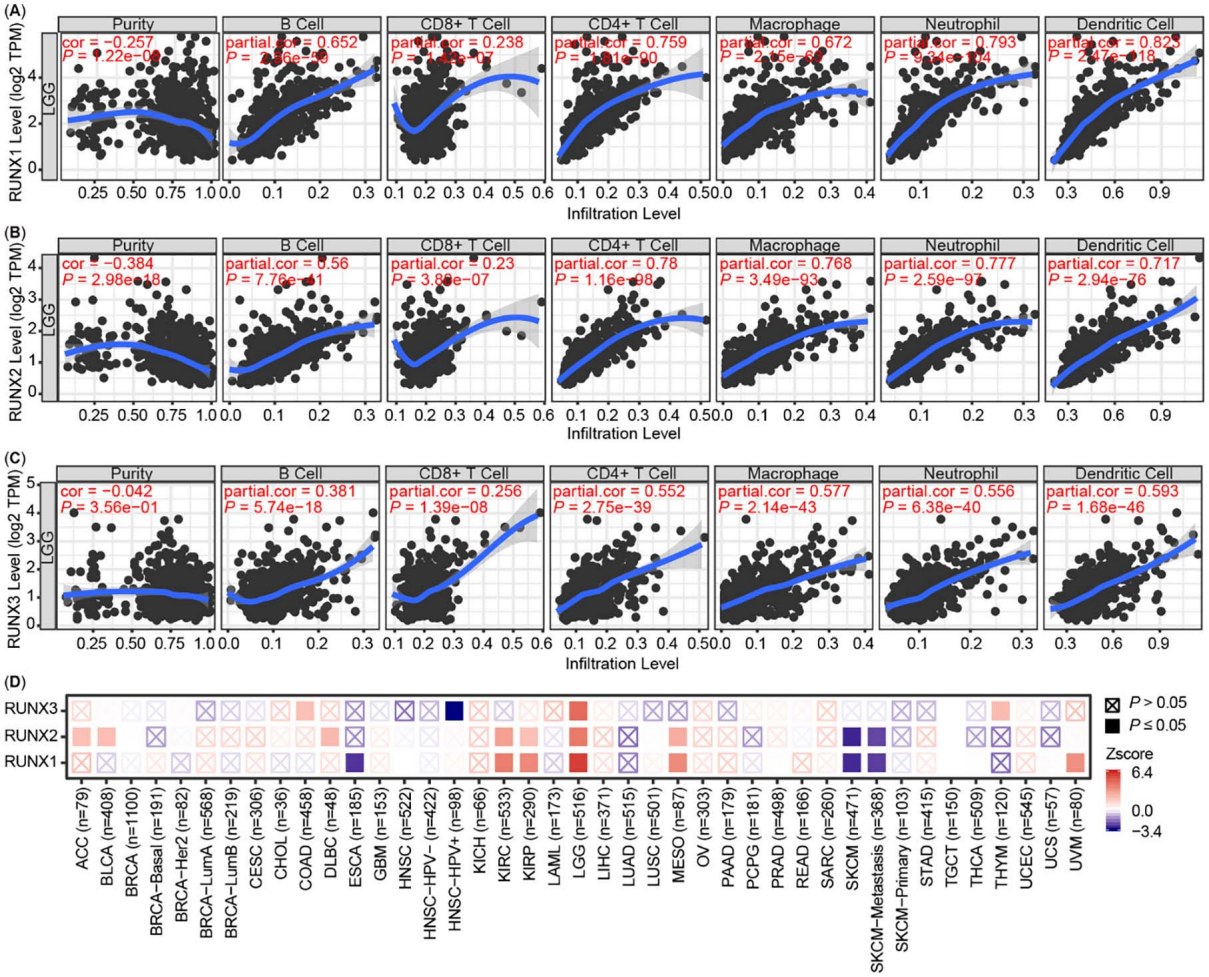
**Correlation of Runt-related transcription factors** **(RUNXs) expression with immune infiltration and prognosis in LGG.** (A)–(C) The correlation between the abundance of immune cells (B cell, CD8^+^ T cell, CD4^+^ T cell, macrophage, neutrophil and dendritic cell) and the expression of (A) RUNX1, (B) RUNX2, (C) RUNX3 in the TIMER database and obtained from the TCGA database. (D) The correlations of RUNXs expression and prognosis of LGG patients based on the TIMER database. LGG: Low grade glioma; TCGA: The Cancer Genome Atlas.

RUNX1, RUNX2, and RUNX3 were all highly correlated with immune cell infiltration in the HNSC, KIRC, LGG, liver hepatocellular carcinoma (LIHC), and prostate adenocarcinoma (PRAD) (Figure S2A–S2C). The relationship was most evident between RUNXs expression and immune infiltration in LGG, as shown in Figure 2A–2C. To explore the relationship among RUNXs expression, immune infiltration, and prognosis, the clinical relevance of RUNXs expression across diverse cancer types was further analyzed by TIMER 2.0, and the prognosis-related heat maps are shown in Figure 2D, the prognosis is cumulative survival and the main evaluation parameters including age, gender, race, stage, and purity. We further used the TIMER database to generate Kaplan–Meier plots in HNSC, KIRC, LGG, LIHC, and PRAD cancer types (Figure S3A–S3C). Our results revealed only B cell infiltration (*P* ═ 0.045) was significantly correlated with HNSC prognosis. RUNX1 expression (*P* < 0.001) and RUNX2 expression (*P* ═ 0.007) were significantly correlated with KIRC prognosis. There was no significant correlation between immune infiltration and KIRC prognosis. Immune infiltration and RUNXs were not correlated with LIHC and PRAD prognoses. However, surprisingly, RUNX1 (*P* < 0.001), RUNX2 (*P* ═ 0.002), and RUNX3 (*P* < 0.001) were highly correlated with the LGG prognosis.

### RUNXs are highly correlated with the progress and prognosis of LGG

**Figure 3.**
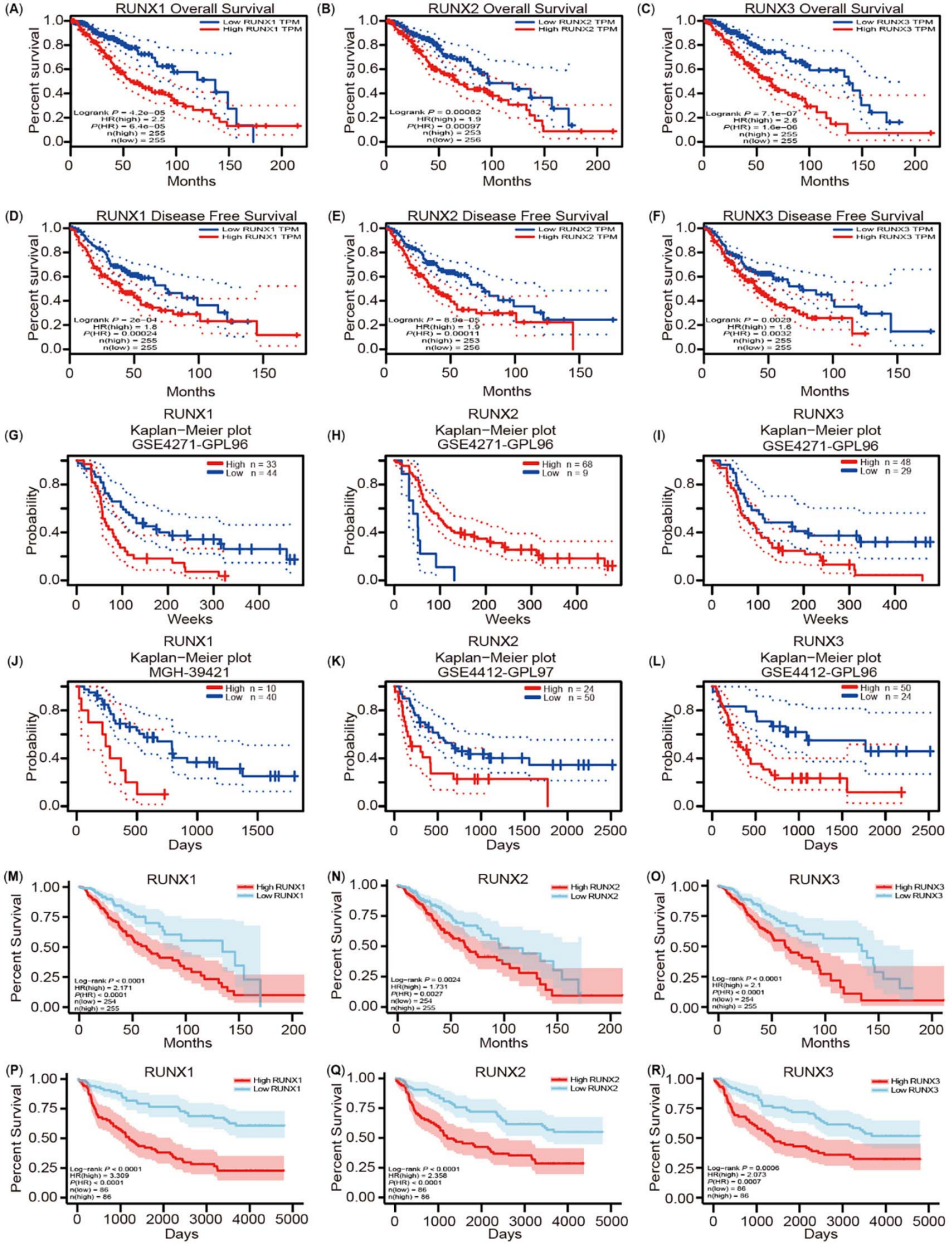
**Correlation between Runt-related transcription factors** **(RUNXs) expression and prognosis of low grade glioma (LGG) patients.** (A)–(F) The expression of RUNXs related to overall survival (OS), disease-free survival (DFS) in the GEPIA database; (G)–(L) Correlation between RUNXs expression and prognosis of LGG cancer in the PrognoScan database; (M)–(O) Correlation between RUNXs expression and OS of LGG based on the The Cancer Genome Atlas (TCGA) database; (P)–(R) Correlation between RUNXs expression and OS of LGG based on the Chinese Glioma Genome Atlas (CGGA) database.

To further explore the significance of RUNXs in the progression and prognosis of LGG, we used the GEPIA data to assess how RUNXs expression relates to patient prognosis in LGG. The GEPIA data analysis revealed that their higher expression was closely related to worse prognosis of patients with LGG (RUNX1: OS HR ═ 2.2, *P* ═ 4.2^e-05^; DFS HR ═ 1.8, *P* ═ 2^e-04^. RUNX2: OS HR ═ 1.9, *P* ═ 0.00082; DFS HR ═ 1.9, *P* ═ 8.9^e-05^. RUNX3: OS HR ═ 2.6, *P* ═ 7.1^e-07^; DFS HR ═ 1.6, *P* ═ 0.0029) (Figure 3A–3F). To further consolidate this result, we used the PrognoScan database and assessed the link between the RUNXs’ expression and cancer patient prognosis. As a result, a significant association was confirmed between the prognosis of LGG and RUNXs (Figure 3G–3L). We further used the TCGA and CGGA database to verify the relationship between RUNXs expression and the prognosis of LGG (Figure 3M–3R). These results clearly demonstrated that a high expression of RUNXs is closely associated with poorer outcomes of LGG.

**Figure 4.**
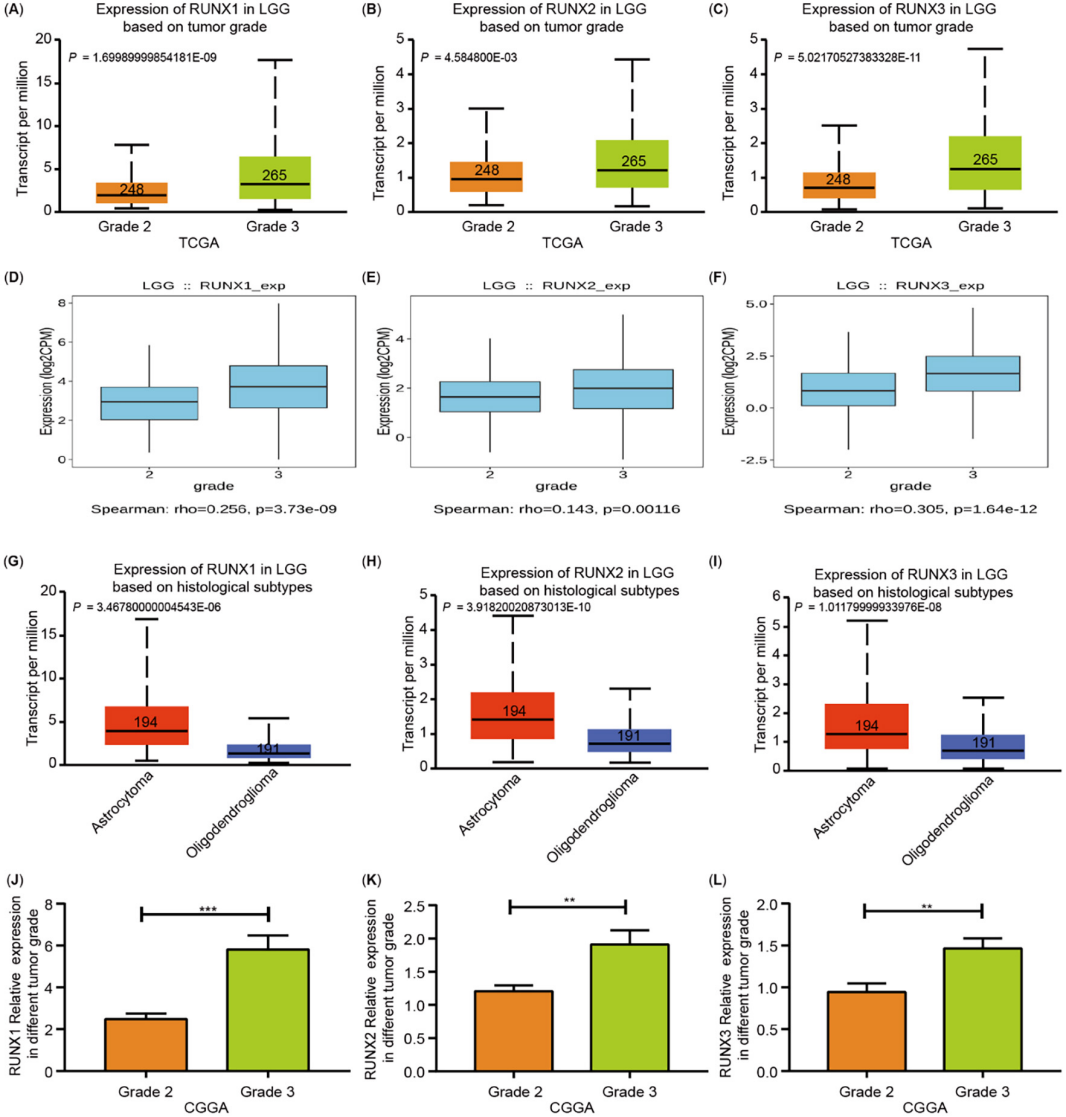
**Runt-related transcription factors** **(RUNXs) transcription in subgroups of patients with low grade glioma (LGG).** (A)–(C) The RUNXs transcription levels were significantly elevated in grade 3 gliomas compared with grade 2 gliomas based on the UACLAN database; (D)–(F) The RUNXs transcription levels were significantly elevated in grade 3 gliomas compared with grade 2 gliomas based on the TISIDB database; (G)–(I) The different expression of RUNXs in different histological subtypes of LGG tissues based on the UACLAN database; (J)–(L) The RUNXs transcription levels was significantly elevated in LGG tissues based on tumor grades from the Chinese Glioma Genome Atlas (CGGA) database.

Meanwhile, we also detected a close correlation between the expression of RUNXs and the grades of LGG. All RUNXs were more highly expressed in grade 3 gliomas than in grade 2 gliomas (Figure 4A–4F). According to the 2021 new classification specified by the WHO, adult-type diffuse gliomas can mainly be categorized into three histological subtypes: (1) Astrocytoma, (2) Oligodendroglioma, and (3) Glioblastoma [1]. LGG compose astrocytoma and oligodendroglioma (WHO grades 2 and 3). Of these subtypes, RUNX1, RUNX2, and RUNX3 in astrocytoma is higher than that oligodendroglioma (Figure 4G–4I).

The CGGA cohort analysis verified a positive correlation between RUNXs and the progression of LGG (Figure 4J–4L). Moreover, the analysis of the TISIDB database revealed that RUNX transcription factors were significantly correlated with the immune and molecular subtypes of LGG (Figure S4A–S4F). Therefore, an increase in inflammatory infiltration around LGG signifies the progression and worse prognosis of LGG patients. Accordingly, we speculated that RUNXs may affect the prognosis of LGG by regulating immune infiltrations.

### Analysis of RUNXs-related signaling pathway in LGG

We further analyzed the mRNA sequencing data from 516 LGG patients of the TCGA using the LinkedOmics function module. The high expression of RUNXs implied a poor prognosis of LGG. Therefore, we focused on the signaling pathways enriched by the high expression of RUNX1, RUNX2, and RUNX3.

**Figure 5.**
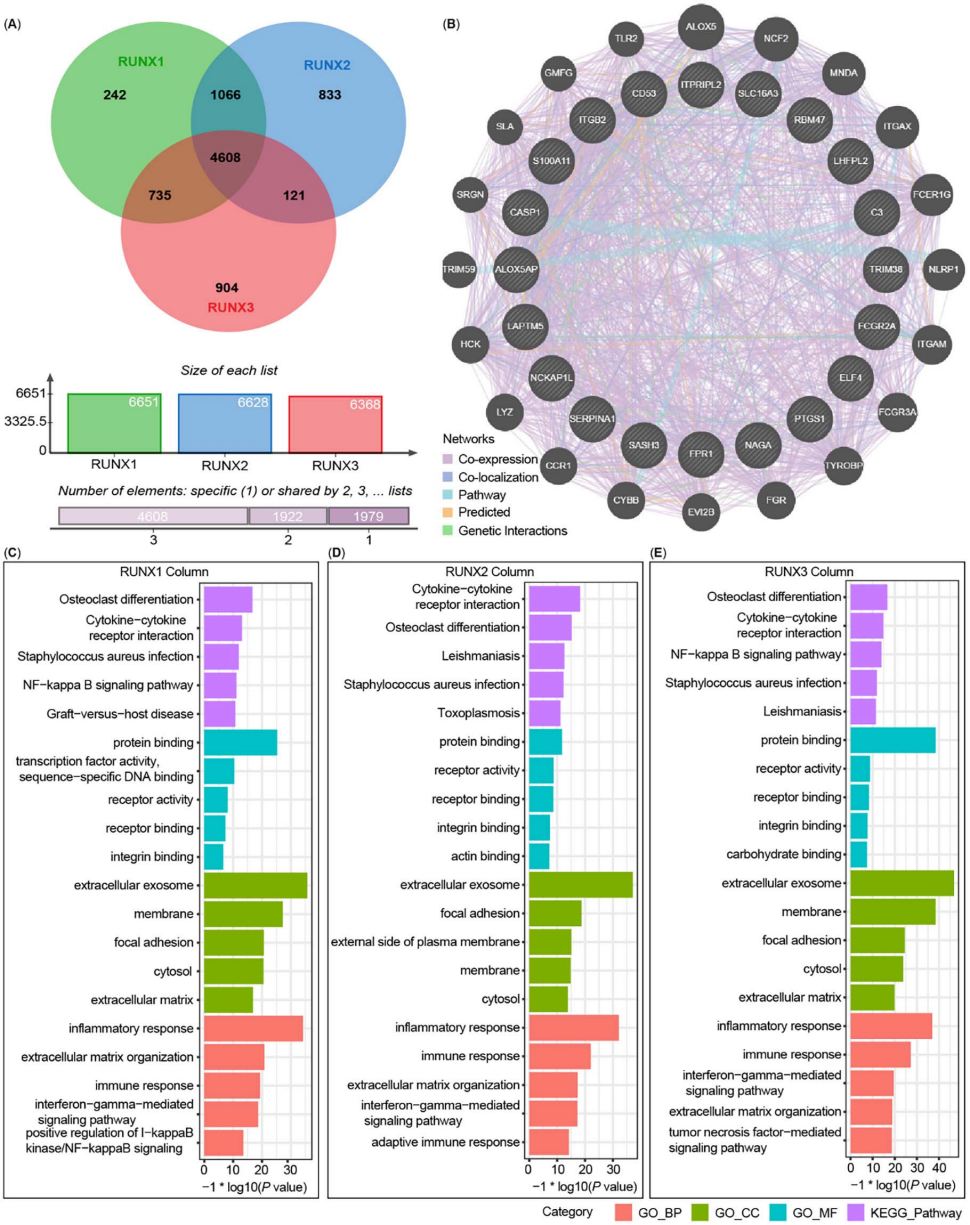
**The related signaling pathway of Runt-related transcription factors** **(RUNXs) in low grade glioma (LGG).** (A) RUNXs positive-related genes were shown in Venn diagrams; (B) The top 20 genes of RUNXs target together were used to construct the protein–protein interaction network via GeneMANIA analysis; (C)–(E) Gene Ontology (GO) and Kyoto Encyclopedia of Genes and Genomes (KEGG) pathway analysis of RUNXs positive-related signatures in LGG.

As depicted in the Venn diagrams (Figure 5A), 6651 genes (green) showed significant positive correlations with RUNX1 (*P* < 0.01, [FDR] < 0.02), 6628 genes (blue) showed significant positive correlations with RUNX2 (*P* < 0.01, [FDR] < 0.02), and 6368 genes (red) showed significant positive correlations with RUNX3 (*P* < 0.01, [FDR] < 0.02). Among these, 4608 genes were enriched by three of them, suggesting a widespread impact of RUNXs on the transcriptome in LGG. The top 20 genes they targeted together were used to construct the PPI network via the GeneMANIA (Figure 5B). There was an evident co-expression among these genes, illustrating the commonness of their targeted genes in performing their functions.

Next, the target genes of RUNX1, RUNX2, and RUNX3 were enriched and analyzed by GO term and KEGG pathway analysis via the Database for Annotation, Visualization, and Integrated Discovery (DAVID) (Figure 5C–5E). GO term analysis revealed that the genes positively related to the expression of RUNXs were mainly located in the area of an inflammatory response, extracellular exosome, and protein binding (Figure 5C–5E). Meanwhile, the osteoclast differentiation and cytokine–cytokine receptor interaction pathways acted as the main enriched pathways through KEGG pathway analysis (Figure 5C–5E). The osteoclast differentiation pathway is one of the most well-known pathways involving RUNXs. Cumulatively, these results imply the participation of these genes in the activation of some receptors or proteins by binding the proteins and receptors, which plays an important regulatory role in an immune or inflammatory response and thus participate in the regulation of TME of LGG.

### Correlation analyses between RUNXs and the molecular expression of immune checkpoints

Tumor immunotherapy is a strategy employed in the treatment of malignant tumors by applying the cytotoxic potential of the human immune system. Immune checkpoints and their ligands are often upregulated in the TME of different malignant tumors, which forms an important obstacle to inducing an effective anti-tumor immune response [38]. In recent years, with the progress in the research on the interaction mechanism between TME and tumor, significant breakthroughs have been made in clinical tumor immunotherapy [39, 40].

**Figure 6.**
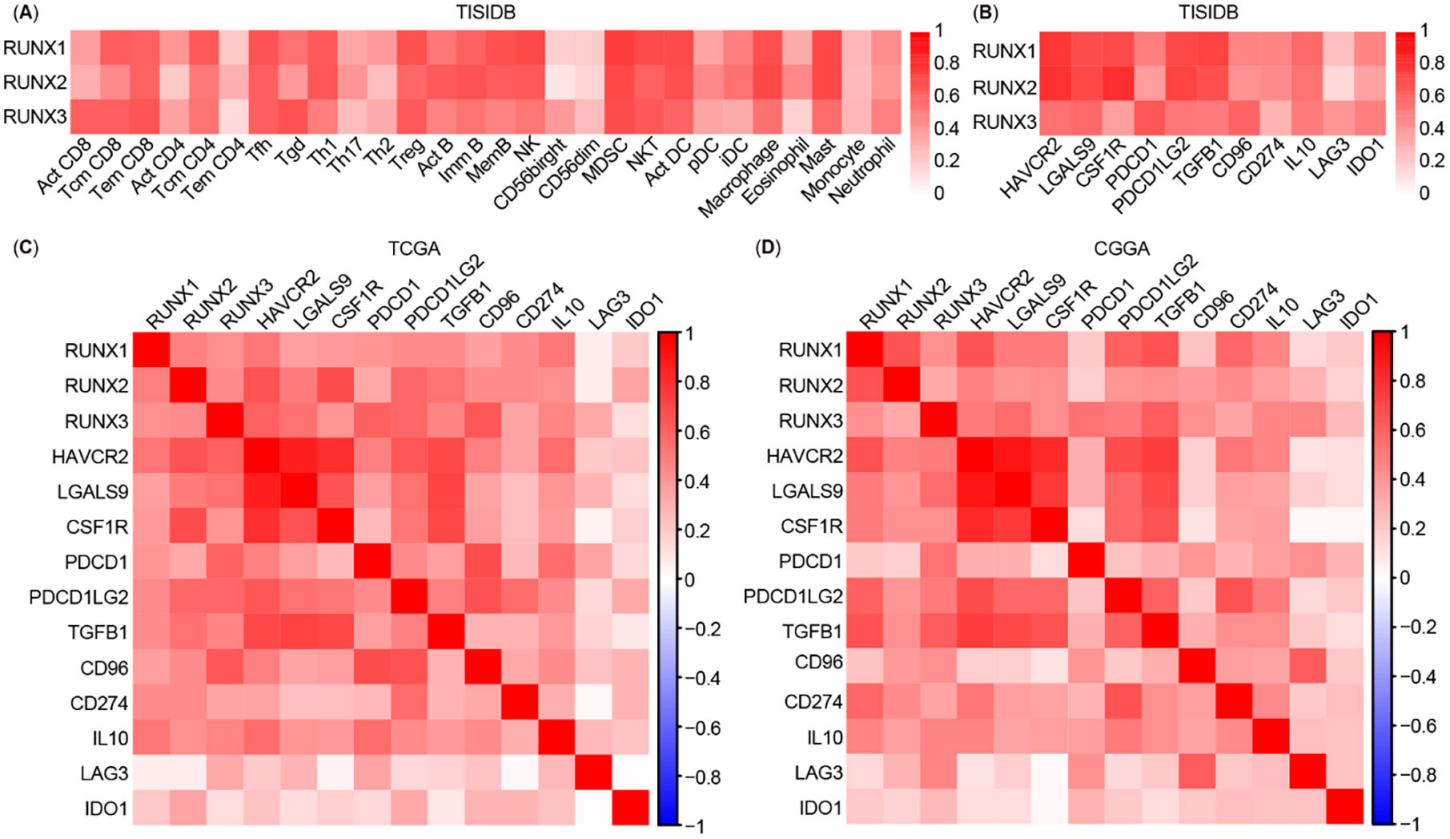
**Correlation analysis between Runt-related transcription factors** **(RUNXs) expression and estimable checkpoint molecules in different databases.** (A) Relations between abundance of tumor-infiltrating lymphocytes and expression of RUNX1, RUNX2, and RUNX3, respectively, in the TISIDB database; (B) Correlation analysis between the expression of RUNXs and estimable checkpoint molecules in the TISIDB database; (C) and (D) Correlation analysis between the expression of RUNXs and estimable checkpoint molecules, and between the estimable checkpoint molecules in The Cancer Genome Atlas (TCGA) and Chinese Glioma Genome Atlas (CGGA) databases, respectively.

We further explored the relationship between the different RUNXs expression and the abundance of tumor-infiltrating lymphocytes (TILs) through TISIDB analysis and found that RUNX1, RUNX2, and RUNX3 were positively correlated with TILs (Figure 6A). Moreover, we recorded a close association between the expression of different RUNXs and most other checkpoint immune molecules, such as HAVCR2, LGALS9, CSF1R, PDCD1, PDCD1LG2, TGFB1, CD96, CD274, IL10, LAG3, and IDO1, which are proven targets of immunotherapy across studies (Figure 6B).

**Figure 7.**
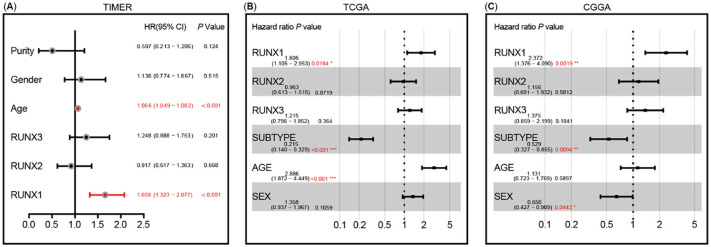
**Multivariate Cox analysis of Runt-related transcription factors** **(RUNXs) expression and other clinical pathological factors.** (A) RUNX1 expression and age are the independent prognostic factors based on TIMER analysis; (B) RUNX1 expression, subtype, and age are the independent prognostic factors based on The Cancer Genome Atlas (TCGA) dataset; (C) RUNX1 expression, subtype, and sex are the independent prognostic factors based on the Chinese Glioma Genome Atlas (CGGA) dataset.

Subsequently, in order to avoid any possible bias, we downloaded the clinical data, gene expression data of patients with LGG from the TCGA database (https://www.cbioportal.org/) [25] and the CGGA databases [26] (http://www.cgga.org.cn/). Patients with grade 2 or 3 LGG defined by WHO and complete clinical and transcriptional data were included for analysis in this study. A similar restricted coexpression pattern was verified through TCGA cohort (Figure 6C, *n* ═ 514) and CGGA cohort (Figure 6D, *n* ═ 182) analyses, and the significant positive correlation between RUNXs and the abovementioned checkpoint immune molecules is illustrated in Figure 6C and 6D.

### RUNX1 represents the family of RUNXs as an independent risk factor for LGG

To explore the comprehensive value of RUNXs in the outcome of LGG patients, we performed RUNXs-related multivariate Cox regression analysis based on LGG.

Multivariate Cox regression analysis of covariates including clinical factors (such as age, gender, and tumor purity) and RUNXs expression using the TIMER database confirmed that age and RUNXs expression are independent prognostic factors (Figure S5A–S5C). However, RUNX2 and RUNX3 lose their independent prognostic value in the presence of RUNX1 (Figure 7A).

As shown in Tables 1–4, multivariate Cox regression analysis of covariates including clinical factors (such as age, gender, and tumor purity), immune infiltrates, and RUNXs expression also revealed that age and RUNX1 expression are independent prognostic factors. Age and macrophage infiltration was identified as another independent prognostic factors that may be affected by the RUNX1 expression. However, RUNX2 and RUNX3 lose their independent prognostic value.

**Table 1:** Multivariate Cox regression analysis of RUNX1 and immune infiltrates

	**Coef**	**HR**	**95% CI_lower**	**95% CI_uper**	***P-*value**
Age	0.057	1.059	1.042	1.076	<0.001***
Sex	0.141	1.151	0.766	1.730	0.499
Purity	−0.418	0.658	0.254	1.705	0.389
B cell	2.130	8.418	0.014	5074.270	0.514
CD8^+^ T cell	3.801	44.739	0.039	51040.985	0.290
CD4^+^ T cell	−2.429	0.088	0.000	393.573	0.571
Macrophage	5.022	151.708	1.950	11802.156	0.024*
Neutrophil	−5.902	0.003	0.000	14.029	0.176
Dendritic	0.187	1.205	0.016	88.695	0.932
RUNX1	0.494	1.639	1.275	2.108	<0.001***

Notes: 469 patients with 111 dying; *P < 0.05, ***P < 0.001. RUNX: Runt-related transcription factors.

**Table 2:** Multivariate Cox regression analysis of RUNX2 and immune infiltrates

	**Coef**	**HR**	**95% CI_lower**	**95% CI_uper**	***P-*value**
Age	0.058	1.060	1.043	1.077	<0.001***
Sex	0.173	1.189	0.793	1.783	0.403
Purity	−0.020	0.980	0.381	2.518	0.967
B cell	2.189	8.926	0.013	6289.716	0.513
CD8^+^ T cell	4.588	98.317	0.082	118311.945	0.205
CD4^+^ T cell	−1.412	0.244	0.000	1733.506	0.755
Macrophage	5.115	166.478	2.550	10868.794	0.016*
Neutrophil	−5.165	0.006	0.000	23.712	0.224
Dendritic	1.905	6.721	0.100	451.470	0.375
RUNX2	0.062	1.064	0.595	1.904	0.834

Notes: 469 patients with 111 dying; *P < 0.05, ***P < 0.001. RUNX: Runt-related transcription factors.

**Table 3:** Multivariate Cox regression analysis of RUNX3 and immune infiltrates

	**Coef**	**HR**	**95% CI_lower**	**95% CI_uper**	***P-*value**
Age	0.058	1.059	1.042	1.077	<0.001***
Sex	0.162	1.176	0.783	1.765	0.434
Purity	−0.083	0.921	0.353	2.403	0.866
B cell	2.800	16.444	0.020	13383.927	0.413
CD8^+^ T cell	4.627	102.166	0.101	103028.780	0.190
CD4^+^ T cell	−0.381	0.683	0.000	2535.974	0.928
Macrophage	4.657	105.273	1.447	7657.663	0.033*
Neutrophil	−5.528	0.004	0.000	17.150	0.195
Dendritic	1.318	3.737	0.043	323.516	0.562
RUNX3	0.167	1.182	0.780	1.792	0.431

Notes: 469 patients with 111 dying; *P < 0.05, ***P < 0.001. RUNX: Runt-related transcription factors.

**Table 4:** Multivariate Cox regression analysis of RUNX family and immune infiltrates

	**Coef**	**HR**	**95% CI_lower**	**95% CI_uper**	***P-*value**
Age	0.057	1.058	1.041	1.076	<0.001***
Sex	0.134	1.144	0.759	1.722	0.521
Purity	−0.437	0.646	0.247	1.691	0.374
B cell	1.954	7.055	0.007	7261.030	0.581
CD8^+^ T cell	4.152	63.547	0.047	85284.250	0.259
CD4^+^ T cell	−1.227	0.293	0.000	2195.897	0.788
Macrophage	5.165	175.060	1.667	18388.167	0.030*
Neutrophil	−6.223	0.002	0.000	10.576	0.155
Dendritic	0.042	1.043	0.012	89.253	0.985
RUNX1	0.504	1.656	1.280	2.143	<0.001***
RUNX2	−0.181	0.834	0.471	1.477	0.535
RUNX3	0.072	1.074	0.705	1.637	0.739

Notes: 469 patients with 111 dying; *P < 0.05, ***P < 0.001. RUNX: Runt-related transcription factors.

To further verify the results of the TIMER database, we employed TCGA and CCGA databases for multivariate Cox regression analysis. Multivariate regression analysis of TCGA data revealed that age, subtype, and the expressions of RUNX1 and RUNX3 could act as independent prognostic factors of LGG, but the RUNX2 expression did not have an independent prognostic value. However, when RUNX1 and RUNX3 coexisted, RUNX3 lost its independent prognostic value (Figure S5D–S5F and Figure 7B). Multivariate regression analysis of CGGA data reached a similar conclusion (Figure S5G–S5I and Figure 7C).

In summary, among the three RUNXs, RUNX1 can be applied as an independent risk predictor for LGG patients’ outcomes, representing the comprehensive risk assessment of RUNXs, and as a potential marker for immune infiltration, progression, and prognosis in patients with LGG.

### RUNX1 may be involved in the progression and immune infiltration of LGG by regulating interferon-gamma receptor 2

**Figure 8.**
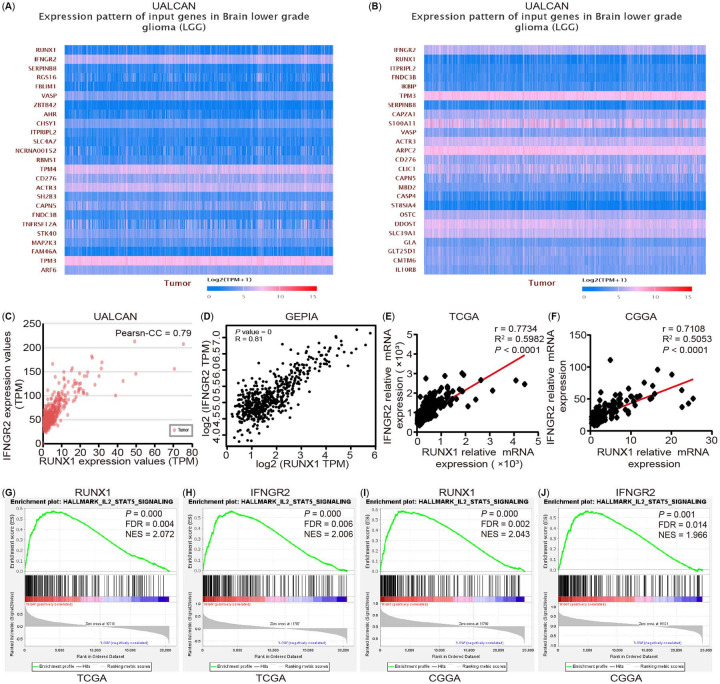
**The analysis of correlation between RUNX1 and IFNGR2 and their related pathway in LGG.** (A) IFNGR2 is the highest correlation gene with RUNX1 transcript level by using RUNX1 as the dominant factor analysis based on the UALCAN database; (B) RUNX1 is the gene with the highest correlation with IFNGR2 transcript level by using IFNGR2 as the dominant factor analysis based on the UALCAN database; (C) and (D) The correlation between RUNX1 and IFNGR2 in cancer tissues in the UACLAN and GEPIA analysis, respectively; (E) and (F) The correlation between RUNX1 and IFNGR2 mRNA expression in cancer tissues from the TCGA and CGGA datasets; (G)–(J) Enrichment plots of GSEA indicate that the gene signatures of IL2-STAT5 pathway were significantly enriched in high RUNX1 (G) and (I)—and IFNGR2 (H) and (J)—expressing LGG specimens based on the TCGA and CGGA datasets, respectively. LGG: Low grade glioma; TCGA: The Cancer Genome Atlas; CGGA: Chinese Glioma Genome Atlas; FDR: False discovery rate; NES: Normalized enrichment score; RUNX: Runt-related transcription factors.

To explore the role of RUNX1 in tumor progression and the immune response of LGG, we explored the highest correlation gene with RUNX1 transcript level through UALCAN analysis and identified IFNGR2 as the target gene. Moreover, we found that RUNX1 had the highest correlation with the IFNGR2 transcript level by using IFNGR2 as the dominant factor analysis (Figure 8A and 8B). Furthermore, Pearson’s correlation coefficient was found to be 0.79 (Figure 8C). The GEPIA database analysis demonstrated a significant positive correlation between RUNX1 and IFNGR2 expression (Figure 8D). Gene analysis revealed that the expression trend of IFNGR2 in different grades and histological types was highly consistent with the model of RUNX1 and that its expression trend in LGG immune and molecular subtypes was also consistent with RUNX1 (Figure S6A–S6E). In addition, the outcome of LGG patients with a high expression of IFNGR2 was worse (Figure S6F–S6K). In addition, a positive correlation was noted between RUNX1 and IFNGR2 expression in LGG, as verified with reference to the TCGA and CGGA cohort analysis (Figure 8E and 8F). The GSEA pathway enrichment analysis revealed that RUNX1 and IFNGR2 shared common features in the IL2-STAT5 pathway (Figure 8G–8J).

Past studies have reported that the upregulation of STATs signaling pathways can promote cell proliferation, migration, invasion, inhibit cell apoptosis, and participation in dysregulated immune surveillance [41]. In recent years, there is increasing evidence that STAT5 is involved in tumor growth, metastasis, and occurrence of anti-tumor drug resistance [42]. When the related ligands of this pathway, including interleukin-2 (IL-2) and IL-3, bind to their respective receptors, STAT5 gets activated, resulting in dimerization or multimerization, followed by the further activation of JAKs and other related pathways [43, 44] and their participation in regulating their target genes, such as MCL1, BCL-2, CyclinD, MMP9, and PIM-1 [42].

**Figure 9.**
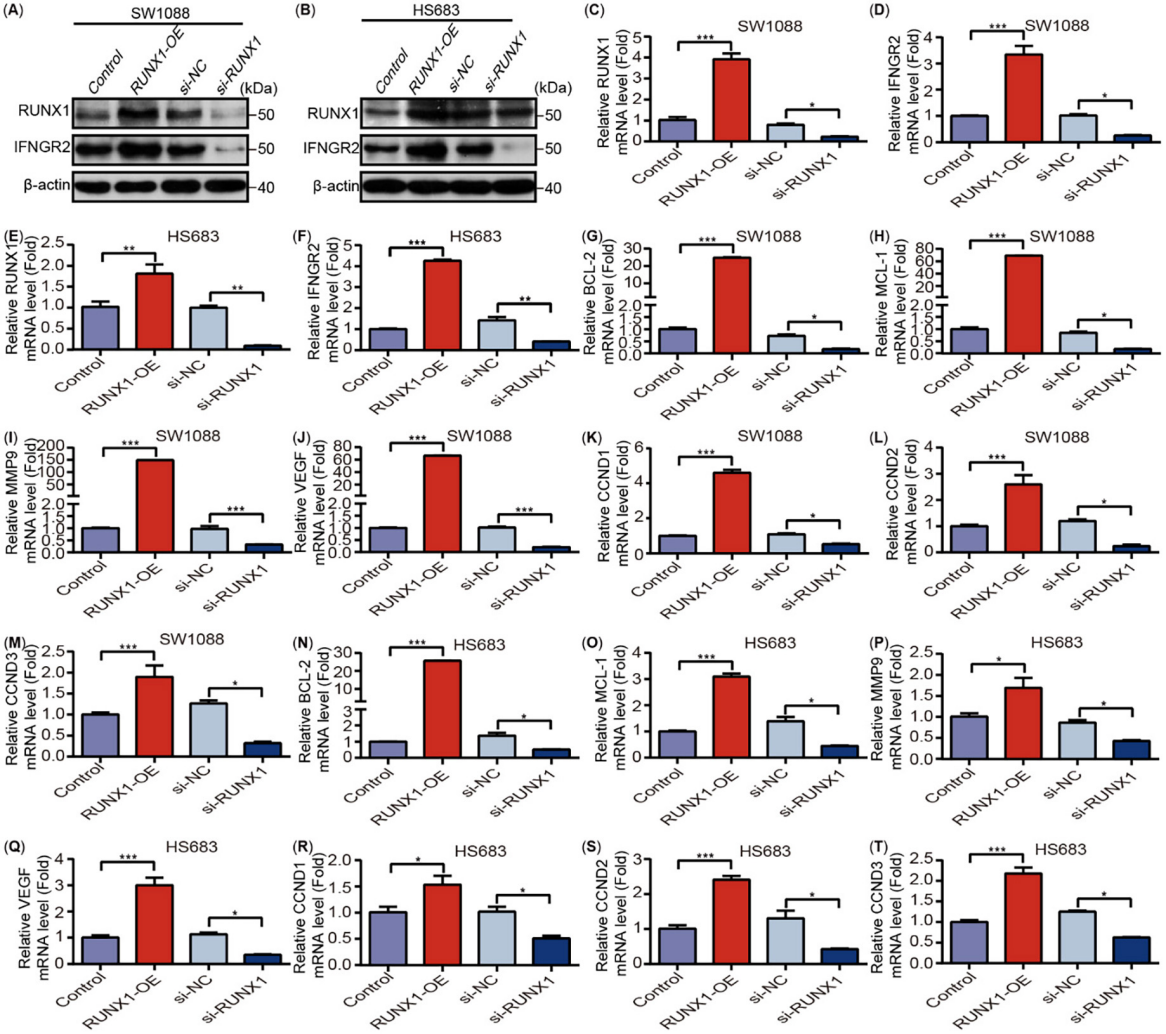
**RUNX1 can regulate the IFNGR2 expression in human LGG cell lines.** (A) and (B) Overexpression or knockdown of RUNX1 could increase or inhibit the IFNGR2 expression in SW1088 and HS683 cells; (C)–(F) Relative mRNA expression of RUNX1, IFNGR2 in SW1088 and HS683 cells (*n* ═ 3); (G)–(T) Relative mRNA expression of BCL-2, MCL-1, MMP9, VEGF, CCND1, CCND2, CCND3 in SW1088 and HS683 cells (*n* ═ 3). Data are shown as presented as mean ± SEM; ^*^P < 0.05, ^**^P < 0.01, ^***^P < 0.001; OE: Overexpression; NC: Negative control; si: Short interfering; IFNGR2: Interferon-gamma receptor 2; LGG: Low grade glioma; RUNX: Runt-related transcription factors.

Accordingly, we examined the IFNGR2 protein expression and mRNA expression after overexpression and knockdown of RUNX1 using two LGG cell lines, namely, SW1088 and HS683, and found that RUNX1 could regulate the expression and transcription of IFNGR2 (Figure 9A–9F). We also examined the related target genes expression of STAT5 signaling, BCL-2, MCL1, MMP9, VEGF, and Cyclin D1, 2, 3 (Figure 9G–9T) and performed CCK-8 proliferation, flow cytometry cell cycle and apoptosis, cell migration, and invasion assays (Figure S7A–S7R). These results confirmed that RUNX1 could monitor the expression of IFNGR2 and participate in the LGG progression by regulating the STAT5 signaling pathway. In turn, it affected the TME of LGG and its response to immunotherapy to a certain extent, although the exact underlying mechanism warrants further experimental verification.

### RUNX1 and INFGR2 are associated with subtype classification and immune infiltration characteristics of LGG

Clinically, LGG patients can be classified into IDH WT and IDH mutant-type according to the characteristics of IDH, and the IDH mutations were associated with prolonged overall LGG survival [45, 46].

**Figure 10.**
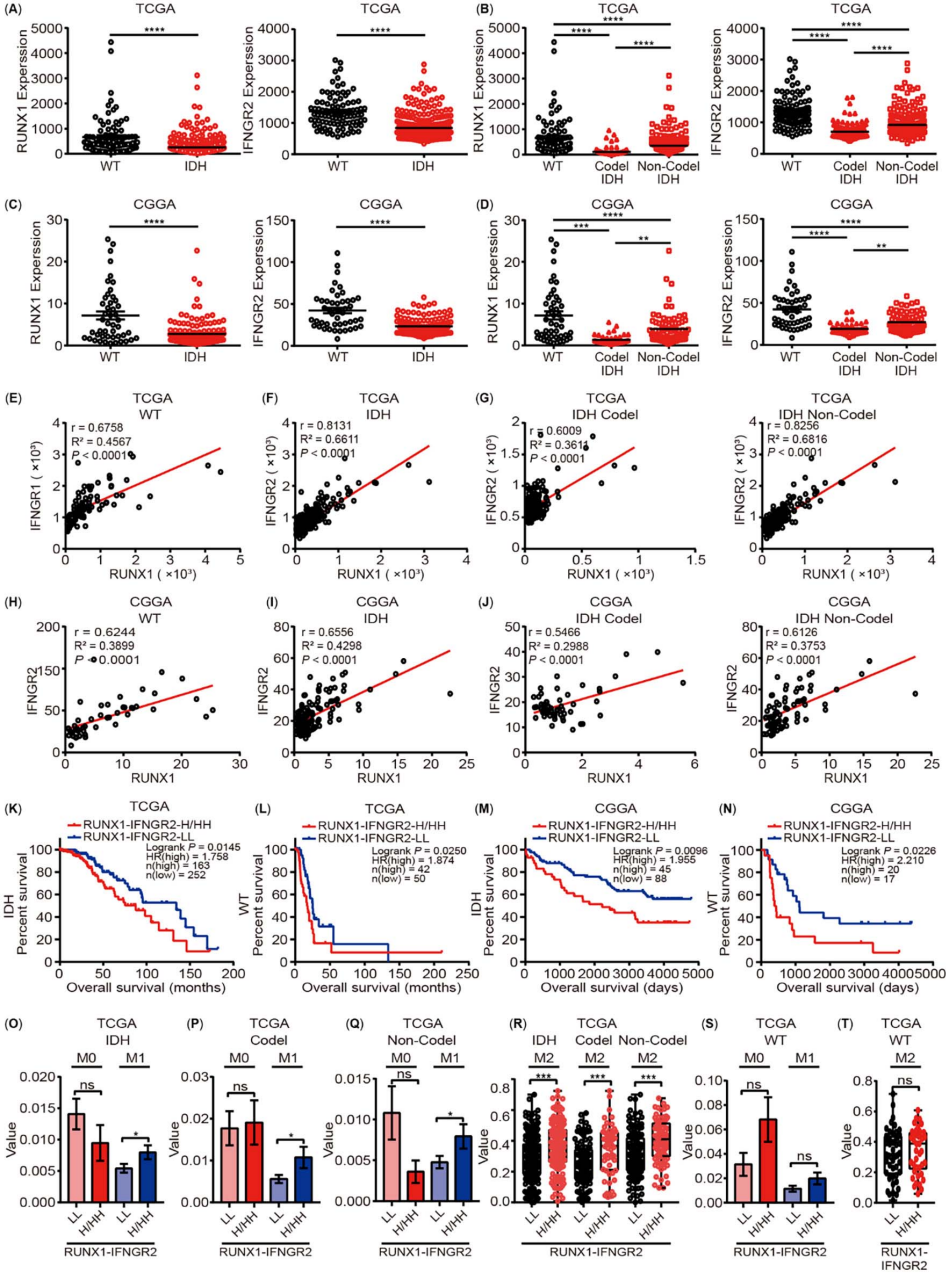
**RUNX1 and INFGR2 are associated with subtype classification and immune infiltration characteristics of LGG.** (A)–(D) The expression of RUNX1 and INFGR2 in LGG tissues based on IDH wild-type (WT) and IDH mutant type with codel or non-codel subtypes from the TCGA and CGGA databases, respectively; (E)–(J) Correlation analysis of the expression of RUNX1 and INFGR2 based on IDH WT and IDH mutant type with codel or non-codel subtypes from the TCGA and CGGA databases, respectively; (K)–(N) The prognostic model analysis by the expression of RUNX1 and IFNGR2 based on IDH WT and IDH mutant type from the TCGA and CGGA databases, respectively; (O)–(R) The M1 and M2 macrophage infiltration was markedly higher in the RUNX1 and/or IFNGR2 overexpression group based on IDH mutant type and IDH mutation with codel or non-codel subtypes from the TCGA database; (S) and (T) The M1 and M2 macrophage infiltration was no significant difference between groups of different expression patterns of RUNX2 and IFNGR2 based on IDH WT subtype from the TCGA database. RUNX: Runt-related transcription factors; LGG: Low grade glioma; TCGA: The Cancer Genome Atlas; CGGA: Chinese Glioma Genome Atlas; INFGR2: Interferon-gamma receptor 2. Data are shown as presented as mean ± SEM; ^*^P < 0.05, ^**^P < 0.01, ^***^P< 0.001. ^****^P< 0.0001.

It has been reported that RUNX1 and its target gene REXO2 are upregulated in the IDH WT subgroup, which is related to the poor prognosis of IDH WT LGG [47]. Our study results showed that RUNX1 could participate in LGG progression by regulating IFNGR2 and possibly participates in the TME regulation of LGG. To further clarify the interaction between RUNX1 and IFNGR2 in LGG and study their effects on LGG progression and subtype differentiation, we performed gene expression and correlation analyses of RUNX1 and IFNGR2 in WT, IDH mutation, and both codel and non-codel subtypes of IDH mutation with reference to the TCGA and CGGA data study cohorts. The results revealed that, in the TCGA cohort, when compared with the WT subtype, the expression levels of RUNX1 and IFNGR2 in the IDH subtype were significantly lower (Figure 10A), while, in the IDH subtype, the expression levels of RUNX1 and IFNGR2 in the codel subtype were lower than in the noncodel subtype (Figure 10B). The analysis results of CGGA cohort also confirmed this conclusion of the TCGA data cohort (Figure 10C and 10D). Meanwhile, further analysis revealed that RUNX1 and IFNGR2 were significantly correlated in the TCGA cohort (Figure 10E–10G), while the expression correlation between RUNX1 and IFNGR2 was higher in the IDH subtype when compared to that in the WT subtype (Figure 10E and 10F). In the IDH subtype, the expression correlation between them was significantly higher in the noncodel subtype than in the codel subtype (Figure 10G). The results of the CGGA data cohort were consistent with those of the TCGA data cohort (Figure 10H–10J). As a result, we further analyzed the correlation between different expression patterns of RUNX1 and IFNGR2 in the WT and IDH subtypes of TCGA and CGGA data cohorts and patient prognosis, suggesting that the simultaneous low expression of RUNX1 and IFNGR2 was associated with better patient prognosis when compared with a high expression of RUNX1 and/or IFNGR2 (Figure 10K–10N).

The degree of immune infiltration in different subtypes of LGG and the prognosis of corresponding patients were different [48]. Past studies have indicated that macrophages, especially M2 macrophages, play a key regulatory role in the progression of glioma [49]. Previously, among different immune infiltrating cells, we found that macrophages can act as an independent prognostic factor in LGG patients and are affected by RUNXs to a certain extent (Tables 1–4). Therefore, we further analyzed the correlation between the different expression patterns of RUNX1 and IFNGR2 as well as macrophage infiltration in different subtypes through the TCGA research cohort. Our results suggested that compared with the low expression group, the infiltration of M1 and M2 macrophages in the high expression group of RUNX1 and/or IFNGR2 was significantly increased in the IDH mutant subtypes (Figure 10O–10R); this phenomenon was not determined in the WT group (Figure 10S and 10T). The abovementioned evidence revealed that the interaction between RUNX1 and IFNGR2 was likely to play a key role in the differentiation of different subtypes of LGG, especially for the efficacy and prognosis of immunotherapy in patients with IDH mutant subtypes, which provides a potential target for the further refinement of different subtypes of LGG in a clinical setting.

## Discussion

The conventional method of tumor treatment involved killing the cancer cells “with the help of external forces” through surgery, radiotherapy, and chemotherapy, while tumor immunotherapy was employed to “attack” cancer cells from within by awakening the autoimmunity system. More emphasis was placed on the key role of the surrounding environment in the occurrence, development, and metastasis of cancer [50, 51]. TME is a unique environment formed by several interactions between tumor cells and the surrounding host cells and their secretions; TME not only plays a key role in tumorigenesis but also in promoting tumor progression [52, 53]. The TME of a solid tumor is heterogeneous and complex; it is composed of cancer cells, interstitial cells, and immune cells [51]. Inflammation plays a decisive role at different stages of tumor occurrence, development, malignant transformation, invasion, and metastasis, as well as affects immune monitoring and treatment response. As a result, there exists a close interaction between the immune cells infiltrating the tumor and the progression of cancer cells [54].

Transcription factors control chromatin and transcription by identifying specific DNA sequences, which together form a complex system that guides the expression of a genome. They form the basis of human physiology, diseases, and mutations, and participate in the occurrence and development of cancer and other diseases [55], accounting for approximately 20% of all oncogenes identified to date [56]. RUNXs belong to a family of metazoan transcription factors, and there are three main RUNX in mammals (namely, RUNX1, RUNX2, and RUNX3), which are the major regulators of development and often deregulated in human cancers [16]. Different RUNXs act as oncogenes or suppressors in different tumors [57] as well as play an important role in the development of immune cells [17, 18]. As is already well established, immune infiltration cells of the TME participate in the tumor progression through interaction with tumor cells [19], although the roles of different RUNXs in the TME are unclear. Therefore, a comprehensive understanding of RUNXs in human cancers is highly warranted.

In the present study, we first performed a pan-cancer analysis of the expression of RUNXs and then demonstrated that RUNX1, RUNX2, and RUNX3 are highly expressed in various cancers. We also verified the different clinical and prognostic roles of RUNX1, RUNX2, and RUNX3 mRNA expressions as well as the correlation between them and immune infiltration in different solid tumors. For the first time in literature, we have reported a significant positive correlation between RUNXs and immune infiltration in diverse cancers. Immune infiltration analyses suggested that RUNX1, RUNX2, and RUNX3 all showed a strong positive correlation with immune infiltration in LGG, but a significant negative correlation with the prognosis of patients with LGG.

LGG includes grade 2 and 3 gliomas, accounting for 43.2% of primary intracranial gliomas [3, 58]. Without intervention, most LGGs gradually progress to high-grade gliomas (WHO grade 4, such as, glioblastoma, IDH-WT; astrocytoma, IDH-mutant). Therefore, we performed expression analysis of different RUNXs in different grades of LGG and demonstrated that, with an increase in grades, the expression of RUNX1, RUNX2, and RUNX3 increased and significant differences were noticeable in the expression of RUNXs among different histological subtypes.

It has been demonstrated that the immunological characteristics of the TME vary significantly among different subtypes of LGG [48]. The associated analysis of RUNXs in LGG suggests that RUNX1, RUNX2, and RUNX3 showed high similarity in the positive-related genes. Moreover, pathway enrichment analysis of the genes positively associated with RUNX1, RUNX2, and RUNX3 was significantly enriched in the inflammatory response and immune response of biological processes. Immunotherapy, including antibodies, chimeric antigen receptor T cells, and immune checkpoint inhibitors (ICIs), has been highly successful in several extracranial tumors [59, 60], which provides a solid rationale for its application in glioma. The interaction between tumor cells and TME determines the effect of immunotherapy [61]. Moreover, different immunological characteristics of the TME often elicit different responses from tumors to immunotherapy [62, 63]; therefore, refining the classification of TME is the key to optimizing the efficacy of immunotherapy. At present, the primary available treatment for LGG is still surgical resection. The combination of radiotherapy and temozolomide chemotherapy is still a first-line adjuvant treatment strategy [64] but the results of treatment vary widely. In recent years, several effective therapeutic methods combined with immunotherapy have attracted more and more researchers’ attention. Immunocheckpoint inhibitors in immunotherapy, such as PD-1/PD-L1, CTLA4, TIM3 and other classic checkpoints, have shown good therapeutic prospects in a variety of tumors and have made significant progress in preclinical and clinical trials [65]. In the present study, we recorded a close association between the expression of different RUNXs and most other checkpoint immune molecules, such as HAVCR2, LGALS9, CSF1R, PDCD1, PDCD1LG2, TGFB1, CD96, CD274, IL10, LAG3, and IDO1. The most well-known immunotherapeutic inhibitor based on the above immune checkpoints is the PD1 (PDCD1), and PDL1 (CD274) inhibitor, such as Pembrolizumab, has different therapeutic effects in different glioma subtypes [66–69]. However, standard therapy with neoadjuvant Pembrolizumab demonstrates significant survival benefits [70]. Another important inhibitor is HAVCR2 (TIM3) inhibitor. HAVCR2 is widely expressed in GBM and IDH-WT gliomas and is able to regulate the inflammatory response after anti-PD-1 treatment [71, 72]. Therefore, the combination of HAVCR2 inhibitors and other immunotherapies has broad application prospects. The antibody against HAVCR2, MBG-453 is in an ongoing phase I trial (NCT03961971). Many inhibitors against other immune checkpoints, including LAG3, IDO, CSF1R, etc., are also in clinical trials [65]. In consideration of the high heterogeneity of LGG and different molecular subtypes have different responses to treatment, the treatment of glioma much relying on molecular biomarkers as criteria of diagnosis and classification. With the advent of many targeted therapeutic options, the search for biomarkers for specific targeted therapies is becoming increasingly important.

Although the LGG grade is relatively low, its recurrence rate is high and it is easy to transform to a higher grade [73]. The prognosis of LGG is affected by factors such as age, functional status score, tumor size, location, whether or not to cross the midline, the degree of neurological impairment, and the scope of resection, among other factors [74, 75]. The importance of the glioma gene-phenotype was emphasized in the 2021 WHO brain tumor classification [1]. In addition, some studies have demonstrated that the infiltration of B cells, T cells, macrophages, neutrophils, and dendritic cells in the TME is an adverse factor that affects the prognosis of patients with LGG [7]. This observation suggests that the gene-phenotype and immune infiltration in the TME are important for the prognosis of patients with LGG.

Our study findings revealed that RUNX1 and macrophages can act as independent prognostic factors of LGG through multivariate Cox regression analysis, and there exists an obvious positive correlation between RUNX1 and IFNGR2. In response to immune monitoring, multiple immune cells were found to functionally migrate to the tumor sites and trigger anti-tumor immunity through the release of a series of cytotoxic factors, such as interferon (IFN)-γ, tumor necrosis factor (TNF)-α and CD95 ligand (FasL), and TNF-related apoptosis-inducing ligand (TRAIL) [76–78]. In addition, a bidirectional function of IFN-γ was recorded in anti-tumor immunity. IFN-γ binds IFNGR1 and IFNGR2, which act on some relevant downstream signal pathways. However, IFN-γ can promote T cell apoptosis and induce the expression of key negative regulatory molecules, such as PD-L1 and indoleamine-2,3-dioxygenase (IDO), during in vivo immune response [79–81]. After the GSEA pathway enrichment analysis, the IL2-STAT5 pathway was enriched by the higher expression of RUNX1 and IFNGR2, which were involved in cell proliferation, migration, invasion, and tumor immunity. Further cell experiments revealed that RUNX1 could regulate the transcription and expression of IFNGR2 as well as participate in glioma cell proliferation, cycle, apoptosis, invasion, and migration, which may be realized through the IL2-STAT5 pathway.

LGG is highly heterogeneous, existing with multiple subtypes. It has also been reported that IDH mutant type LGG presents with a better prognosis when compared with the IDH wild subtypes [1]. Significant differences were also noted in the prognosis and immune infiltration characteristics of different mutant subtypes [48]. When compared with the WT subtype, RUNX1 and IFNGR2 showed significantly lower expression in the IDH mutant subtype, albeit the correlation between RUNX1 and IFNGR2 was enhanced, and patients with a high expression of RUNX1 and/or IFNGR2 in the IDH mutant subtype demonstrated worse prognosis and significantly enhanced infiltration of M2 macrophages. However, this result was different from that of the analysis of wild types. Macrophages, especially M2 macrophages, were negatively associated with the survival of glioma patients and positively with the glioma progression [49], which implicated different infiltration characteristics in different LGG subtypes [48]. These results indicate that the interaction between RUNX1 and IFNGR2 can play an extremely critical role in the differentiation of different subtypes of LGG, especially in the differentiation progress of the IDH mutant subtype and the formation of the infiltration characteristics of the TME.

The TME is created by tumors. Immune cells in the TME not only exert anti-tumor effects but also promote tumor growth [19, 82]. Past studies have demonstrated that leukocyte infiltration in tumors, including macrophages and neutrophils, is now recognized as one of the “markers of cancer” [51]. The inhibition of immune cell functions or anti-tumor effector cell is the root cause of tumor immune escape [19]. Therefore, a better understanding of the cellular and molecular mechanisms in the TME can majorly facilitate the prevention of tumor escape [19]. Determining the key molecules that regulate the activity of immune cells in the TME is therefore extremely important for establishing better markers of the TME, improving the efficacy of tumor immunotherapy, and finding more effective immunotherapeutic drugs. Our present results especially provide a potential target for the evaluation of the efficacy and prognosis of immunotherapy in patients with the IDH mutant subtype of LGG and a new high-accuracy immunological biomarker to predict the prognosis of different subtypes of LGG so as to serve as a standard reference for immunotherapy.

## Conclusion

We obtained three RUNX genes (RUNX1, RUNX2, and RUNX3) that were significantly negatively associated with the prognosis of LGG patients. Multivariate Cox regression analysis further established RUNX1 and macrophages as independent prognostic factors for LGG. We also found that RUNX1 can regulate the transcription and expression of IFNGR2 and is involved in the regulation of glioma cell proliferation, cycle, apoptosis, invasion, and migration, and the differentiation process of glioma mutant subtypes. RUNX1 and IFNGR2 can serve as the most important potential therapeutic and prognostic target.

## Supplemental Data

Supplementary data are available at the following link: https://www.bjbms.org/ojs/index.php/bjbms/article/view/8086/2749.
